# Effects of Ramadan Fasting on Serum Amyloid A and Protein Carbonyl Group Levels in Patients With Cardiovascular Diseases

**DOI:** 10.15171/jcvtr.2015.12

**Published:** 2015

**Authors:** Hami Asadi, Ali Akbar Abolfathi, Reza Badalzadeh, Maryam Majidinia, Alireza Yaghoubi, Maryam Asadi, Bahman Yousefi

**Affiliations:** ^1^ Department of Biological Science, Ahar Branch, Islamic Azad University, Ahar, Iran; ^2^ Cardiovascular Research Center, Tabriz University of Medical Sciences, Tabriz, Iran; ^3^ Department of Biochemistry and Clinical Laboratories, Faculty of Medicine, Tabriz University of Medical Sciences, Tabriz, Iran; ^4^ Immunology Research Center, Tabriz University of Medical Sciences, Tabriz, Iran; ^5^ Students’ Research Committee, Tabriz University of Medical Sciences, Tabriz, Iran

**Keywords:** Cardiovascular Diseases, Fasting, Protein Carbonylation, Serum Amyloid-A Protein

## Abstract

***Introduction:*** Serum amyloid-A (SAA) and protein carbonyl group are rigorously related with cardiovascular diseases (CVDs) as a sensitive marker of an acute inflammatory state and as an important index of oxidative stress, respectively. Moreover, diet is one of the main factors that can modify cardiovascular risks. Therefore, this study aimed to investigate the effects of Ramadan fasting on SAA and protein carbonyl group levels in patients with CVDs.

***Methods:*** Twenty-one patients (21 male; mean age 52±9 years old) with CVDs (coronary artery disease, cerebrovascular, or peripheral arterial diseases) were participated in this study. Biochemical parameters were measured in patients 2 days before and 2 days after Ramadan fasting. SAA levels were assessed using enzyme-linked immunosorbent assay and Cayman’s protein carbonyl colorimetric assay was provided for measuring protein carbonyl groups.

***Results:*** According to the findings of the study, post-Ramadan levels of inflammatory biomarker, SAA was decreased significantly in patients with CVDs in comparison with the baseline before-fasting values (16.84±8.20 vs. 24.40±6.72 μg/ml, *P * = 0.021). In addition, Ramadan fasting significantly reduced the levels of protein carbonyl group in patients as compared with those of baseline values (33.08±15.31 vs. 43.65±16.88 nmol/ml, *P * = 0.039).

***Conclusion:*** Ramadan fasting has impressive effects on modulating CVDs by decreasing inflammation and oxidative stress markers. However, to get a clear conclusion with more results, further investigation is warranted.

## Introduction

One of the main rituals of Islam, the religion worshiped by more than one billion people, is fasting during the holy month of Ramadan. In this month, Muslims abstain from eating and drinking from dawn to sunset. Food and fluid intake are occurred in the night and the frequency and quantity of eating, nightly sleep duration, and daily physical activity are usually reduced during Ramadan.^1^ The food habits during Ramadan are not similar to the other months, thus the proportion of fat, protein, and carbohydrate intake can be altered during Ramadan.Previous studies showed that Ramadan fasting has an noticeable effect on blood pressure levels, lipid profiles, and also other cardiovascular disease markers such as serum high sensitivity C-reactive protein (hs-CRP) and homocysteine.^2,3^ The other studies conducted in Turkey and Albania have confirmed a major reduction in hospital admission rates for cardiovascular diseases during the Ramadan month.^4,5^

Cardiovascular disease (CVD) is now the leading cause of death worldwide.^6^ It has been suggested that inflammation is an important pathogenic feature in different clinical settings and accumulating studies demonstrate that oxidative stress also alters many functions of the cardiovascular system.^7^ Overproduction of superoxide and other reactive oxygen species (ROS) seems to occur in conditions such as hypercholesterolemia, hypertension, diabetes, and CVDs.^8^

Serum amyloid-A (SAA) is a proteins family which forms a major component of the acute-phase inflammatory response.^9^ SAA is synthesized by liver in response to inflammation, stress, infection, and injury similar to the C-reactive protein (CRP). As a result, SAA is a sensitive indicator of acute inflammatory state.^10^

In addition, oxidatively modified proteins can act as important biomarkers of oxidative stress. Carbonyl groups might be produced by reactions of proteins with aldehyde products during lipid oxidation or/and with reactive carbonyl compounds formed as a result of reducing sugars or their oxidation compounds with Lys amino acid residues of proteins.^11^ Therefore, the existence of carbonyl groups in proteins has been commonly used as a worthy indicator of ROS-mediated protein oxidation. Consequently, It has been reported that SAA levels, protein oxidation and higher levels of carbonyl group are associated with CVDs.^10,12^

Actually, Ramadan fasting is a radical change in lifestyle for a period of one month that may affect the CVDs and related biomarkers. Thus, the present study was designed to evaluate the effects of Ramadan fasting on SAA and protein carbonyl group levels in patients with CVDs.

## Materials and Methods

### Participants

The study population was consisted of 21 male patients between 41 and 67 years old who had referred to Shahid-Madani Heart hospital in Tabriz in 2013. The women were not included in this study because they do not allow getting fast during some days of their menstrual period through Ramadan month based on the religious rituals of Islam. Diagnosis of CVDs was based on the cardiologist decision. Smoker subjects, patients with any type of acute inflammation (hs-CRP>5), kidney diseases, diabetes, or malignant tumors were excluded from the study. First, All participants were requested to complete a questionnaire form and then necessities were thoroughly explained to them. Dietary pattern of all participants during the Ramadan month was almost the same including high-carbohydrate low-fat diet, vegetables and fruits.

###  Blood Sampling

Peripheral venous blood samples were taken into tubes in the fasting state at 8:00 AM from all subjects 2 days before Ramadan fasting and 2 days after Ramadan. Blood sam­ples were centrifuged at 2500 rpm for 10 minutes, and serum was separated. The prepared samples were stored at −80^°^C until analysis.

###  Measurement of SAA

The serum contents of SAA were assessed by enzyme immunoassay procedure. SAA level was measured using a specific human SAA ELISA kit (Abcam Ltd., Cambridge, UK) according to the manufacturer’s instructions. The absorbance was determined at 450 nm.

### Protein carbonyl group assessment

Serum carbonyl was determined by measuring the protein carbonyl residues using the dinitrophenylhydrazine (DNPH). According to the procedure provided by Cayman’s protein carbonyl colorimetric assay kit (Cayman Chemical, Ann Arbor, USA), the amount of protein-hydrozone product was quantified spectrophotometrically at the wavelength of 360 nm.

###  Statistical analysis

Data were expressed as means ± standard deviation (SD). Pre- and post-Ramadan levels of parameters were compared by paired *t* test. The 𝑃-value below 0.05 was considered statistically significant. The SPSS 16 was used for statistical analysis.

## Results

In the present experiment, the selected patients with CVDs who also got fasting during Ramadan month were assessed for the levels of inflammatory and oxidative stress biomarkers before and after fasting. Demographic and basic information of patients are shown in Table 1. At the beginning of the study, 31 patients were invited to participate in the study, but 7 patients could not follow the requirements of the study (fasted less than 25 days during Ramadan), therefore the remaining were 24 patients from which 3 of them also could not then pursued the study and they went away from the study in succeeding days by themselves. Therefore, pre- and post-Ramadan evaluation was carried out on 21 male patients with the mean age of 52±9 years old. The mean year of history CVDs was 8.0±1.5 years (Table 1).

**Table 1:** Demographic and basic information of patients with CVDs

**Demographic information of patients**	** Values ** ** and items **
Number (final)	21
Sex	Male
Mean age	52±9 years old
Mean age of diseases history	8.0±1.5 years
Nutritional status	High fruits, vegetables, carbohydrate, and low fat
Fasting period	25-30 days during Ramadan
Disease classification	Coronary artery disease, cerebrovascular or peripheral arterial diseases
Excluded risk factors and comorbidity	Smoking, acute inflammation, kidney diseases, malignant tumors, diabetes

### Significant decrease in SAA levels after Ramadan fasting

**Figure 1:**
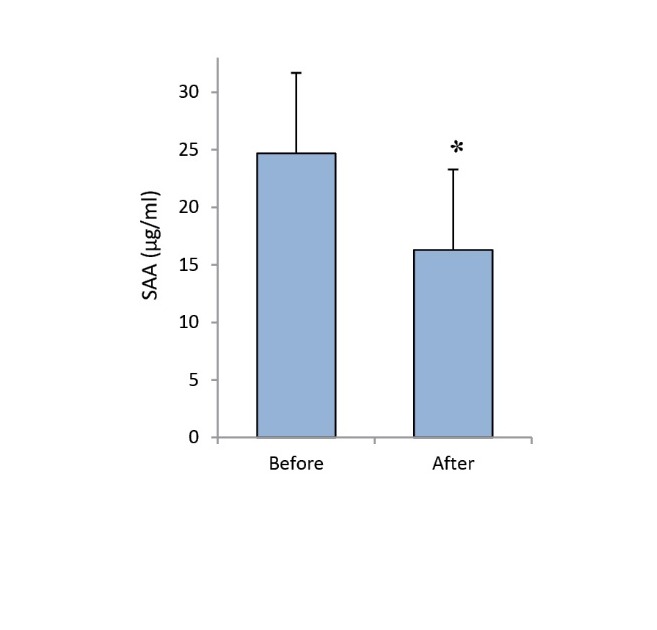
Pre- and post-Ramadan fasting levels of serum amyloid A (SAA), *P *= 0.021in patients with CVDs. Data was represented asmean±SD and experiments performed in triplicates. Paired *t* test was used to compare pre- and post-Ramadan levels. *Significant difference.

This study evaluated the effects of Ramadan fasting on SAA levels as an acute-phase inflammatory protein and useful factor in cardiovascular risk prediction. Our results showed that Ramadan fasting significantly decreased the SAA levels as compared with those of pre-Ramadan values (16.84±8.20 vs. 24.40±6.72 µg/ml, *P *= 0.021, Figure 1).

### Ramadan fasting significantly reduced protein carbonyl group

**Figure 2:**
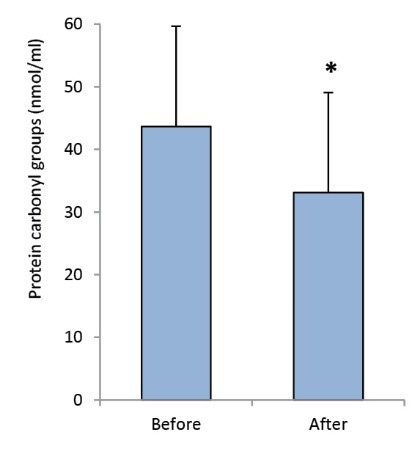
Pre- and post-Ramadan fasting levels of protein carbonyl groups,* P *= 0.039; in patients with CVDs. Data was represented as mean±SD and experiments performed in triplicates. Paired *t* test was used to compare pre- and post-Ramadan levels. *Significant difference.

The presence of carbonyl groups in proteins was determined in patients with CVDs after Ramadan fasting as an indicator of protein oxidation by oxidative stress. Post-Ramadan fasting levels of this biomarker was lower than pre-Ramadan fasting levels, and this difference was statistically significant (33.08±15.31 vs. 43.65±16.88 nmol/ml, *P *= 0.039, Figure 2).

## Discussion

During Ramadan, Muslims appreciative to cease their eating, drinking, smoking and sexual relations from sunrise (*Sahur*) until sunset (*Iftar*). In the remaining hours, they are then allowed to eat. Ramadan fasting provides the best opportunity to evaluate the effects of the prolonged reduction of meal time on body metabolism.^1,13^

In the previous study, we investigated, for the first time, the effects of Ramadan fasting on endothelial function in CVDs.^14^ Our previous results showed that Ramadan fasting have beneficial effects on serum endothelial function markers and can modify cardiovascular risks by decreasing the levels of asymmetric dimethylarginine and malondialdehyde (MDA) and increasing the levels of vascular endothelial growth factor and nitric oxide.

We designed this study to evaluate the effects of Ramadan fasting on the alterations of SSA, and protein carbonyl group (two biomarkers for inflammation and oxidation processes) in patients with CVD. We showed that both biomarkers were significantly lowered during Ramadan fasting in patients. Because a few numbers of patients with CVD get fast thorough Ramadan month, we could not increase the sample population of the study. Therefore, this is one of the limitations of this study.

Laboratory studies have established that inflammation plays an important pathophysiological role in atherogenesis and development of atherosclerotic plaques in coronary arteries and finally CVDs.^15^ This finding was supported by high concentration of hs-CRP in these patients.^16^ The exact mechanism for this association is unknown. SAA as a novel marker of acute inflammatory state, like hs-CRP, has been linked to CVDs. Johnson et al. found higher levels of SAA in female patients with CVDs and concluded a strong independent relationship between SAA and future cardiovascular events, similar to that found for hs-CRP.^17^

Previous studies have indicated that Ramadan fasting decreases oxidative stress and inflammation. Faris et al^18^ carried out a cross-sectional study for the investigation of circulating pro-inflammatory cytokines, and immune cells (monocytes, granulocytes, and lymphocytes) in fasted participants in Ramadan. The pro-inflammatory cytokines IL-1β, IL-6, and TNF-α were significantly lowered after Ramadan fasting. Immune cells were significantly reduced during Ramadan, but within the reference ranges. These results indicate that Ramadan fasting attenuates inflammatory status of the body by suppressing the expression of pro-inflammatory cytokines and decreasing the circulating levels of leukocytes. Weight loss during Ramadan fasting has been attributed also to a decrease in the levels of cytokines and macrophage infiltration due to decrease in the number of monocytes.^18-20^ In another study, Chennaoui et al^21^ showed that IL-6, CRP and homocysteine levels were significantly low during Ramadan in the fasting subjects of both genders when compared to basal values or to volunteers who did not fast. In accord with these findings, the concentration of SAA, as a novel marker for inflammation, was significantly decreased after Ramadan fasting in the present study. This reduced level of SAA in in the serum of investigated patients suggests a beneficial influence of Ramadan fasting on CVD.

On the other hand, the large-scale evidence suggests that oxidative stress is also involved in the pathogenesis of many CVDs.^8,22^ Modification of proteins and production of carbonyl derivatives are the effects of oxidative stress on proteins. Carbonyl protein accumulation is generally assigned not only to oxidative stress but also to disease-derived protein dysfunction.^23^ The effects of fasting on oxidative stress have investigated by different groups by mean of assessing different markers of oxidative stress. Lahdimawan et al^24^ reported that Ramadan altered classically activated macrophage regulation and signaling, reducing macrophage oxidative stress. In addition, Ibrahim and colleagues measured MDA, glutathione, glutathione peroxidase and catalase levels in individuals during Ramadan. Erythrocyte MDA decreased, while the reduction in lipid peroxidative damage enzymes in erythrocytes was slight.^25^ Furthermore, we have reported in our previous study that Ramadan fasting could reduce the MDA levels in healthy subjects and patients with CVD.^14^ In accordance with previous reports, the results of the present study showed the decreased levels of protein carbonylation as an oxidative stress marker in fasted patients. Thus, decreased protein carbonyl levels may lower the negative effects of oxidative stress on proteins.

It is noteworthy to point out that the improvements in serum lipids during Ramadan may have beneficial effects on cardiovascular risk factors. During the acute-phase reaction, SAA associates predominantly with HDL and, therefore, it alters HDL-mediated cholesterol delivery to the cells. This observation may explain its higher concentration in patients with CVD.^11^ Serum triglyceride and HDL-C levels and also TC/HDL-C ratio were markedly improved after Ramadan fasting in comparison to pre-Ramadan. Such improvements could be attributed to both caloric restriction as well as the timing of caloric uptake. Typically, two meals are eaten each day during Ramadan; one light meal just before dawn and the other, which is relatively large, immediately after sunset. Therefore, we hypothesize that decrease in lipid content and increase in HDL levels can reduce fat peroxidation and oxidative stress in fasting states. On the other hand, the high intake of fresh vegetables and fruits rich in antioxidant during Ramadan could be the other reason for reduced lipid peroxidation. However, further study is warranted to get a clear conclusion in this regard.

In conclusion, Ramadan fasting, as a diet intervention can positively modulate cardiovascular risks in patients by decreasing the levels of SAA as a biomarker of inflammation and protein carbonyl group as an important marker of oxidative stress.

## Ethical Issues

The experimental protocols were approved by the ethical committee of Tabriz University of Medical Sciences. Verbal and written consents were obtained from all subjects prior to participation.

## Competing Interests

The authors declare that they have no conflict of interests.
